# Forming a Complex with MHC Class I Molecules Interferes with Mouse CD1d Functional Expression

**DOI:** 10.1371/journal.pone.0072867

**Published:** 2013-08-29

**Authors:** Renukaradhya J. Gourapura, Masood A. Khan, Richard M. Gallo, Daniel Shaji, Jianyun Liu, Randy R. Brutkiewicz

**Affiliations:** Department of Microbiology and Immunology, Indiana University School of Medicine, Indianapolis, Indiana, United States of America; Karolinska Institutet, Sweden

## Abstract

CD1d molecules are structurally similar to MHC class I, but present lipid antigens as opposed to peptides. Here, we show that MHC class I molecules physically associate with (and regulate the functional expression of) mouse CD1d on the surface of cells. Low pH (3.0) acid stripping of MHC class I molecules resulted in increased surface expression of murine CD1d on antigen presenting cells as well as augmented CD1d-mediated antigen presentation to NKT cells. Consistent with the above results, TAP1-/- mice were found to have a higher percentage of type I NKT cells as compared to wild type mice. Moreover, bone marrow-derived dendritic cells from TAP1-/- mice showed increased antigen presentation by CD1d compared to wild type mice. Together, these results suggest that MHC class I molecules can regulate NKT cell function, in part, by masking CD1d.

## Introduction

CD1d constitutes a third antigen (Ag) presenting pathway to contrast with those mediated by major histocompatibility complex (MHC) class I and MHC class II molecules [1,2]. Whereas MHC class I molecules present peptide Ags to T cells, the structurally similar CD1d molecules present a variety of lipids that include normal endogenous glycolipids, glycolipids from marine sponges and bacteria, or tumor-derived phospholipids, glycolipids and non-lipidic molecules [3,4]. Both MHC class I and CD1d molecules are heterodimers comprised of an α heavy chain consisting of three extracellular domains (α_1,_ α_2_ and α_3_) non-covalently associated with β_2_-microglobulin (β_2_-m) [5,6]. However, they differ in their Ag binding groove as it is deeper and more hydrophobic in CD1d molecules than in MHC class I [7,8]. This difference in the Ag binding groove is not surprising, given the structural and chemical differences in the Ags these molecules present—i.e., lipids versus peptides. Another difference between MHC class I and CD1d is their cellular expression. MHC class I molecules are ubiquitously expressed on essentially all nucleated cells, whereas CD1d molecules are present primarily on professional antigen presenting cells (APCs) such as macrophages, dendritic cells and B cells [9–12], although some non-hematopoietic cells such as endothelial cells and hepatocytes can be CD1d^+^ as well [13,14]. Some tumor cells such as lymphomas and leukemias also express CD1d molecules on their surface [12,15].

Several previous reports suggest a link between CD1d and MHC class I. NKT cells express on their surface many of the same receptors as NK cells that are known to interact with MHC class I molecules [16–19]. The expression of CD1d in the thymus is the inverse of that by MHC class I molecules [20]. Furthermore, we found that transporter associated with antigen presentation 1 (TAP1)-deficient mice have higher levels of CD1d in on the surface of macrophages and dendritic cells [21]. Based on these reports, we asked if MHC class I expression could affect the ability of CD1d to be recognized by NKT cells. We report here that MHC class I forms a complex with CD1d, impairing the ability of CD1d to activate NKT cells.

## Materials and Methods

### Mice

Female C57BL/6 wild type (WT) and TAP1-deficient mice were purchased from the Jackson Laboratory (Bar Harbor, ME) and used at 6-8 weeks of age. All procedures were approved by the Institutional Animal Care and Use Committee of the Indiana University School of Medicine (study numbers 2849 and 3636).

### Cell lines, retroviruses and antibodies

Mouse LMTK fibroblasts transfected with *cd1d1* (LMTK-CD1d1) and vector control cells (LMTK-control) have been described previously [22]. These cell lines were cultured in DMEM supplemented with 10% FBS, 2 mM L-glutamine, and 500µg/ml G418. B2MSV40 cells (kindly provided by Dr. S. Tevethia) are murine fibroblasts derived from β2-microglobulin-deficient mice [23]. KT4 cells [24], a murine kidney fibroblast cell line derived from TAP1-deficient mice, were transduced with the pMSCV-puro retrovirus generated using E-86 ecotropic packaging cells (Clontech, Mountain View, CA) expressing the cDNA for murine wild type *cd1d1* (KT4-CD1d1), tail-deleted form [ref [25].] *cd1d1* (KT4-CD1d1TD) or empty vector control (KT4-control) and selected in 2 µg/ml puromycin. The Vα14^+^ (canonical) mouse CD1d-specific NKT cell hybridomas, DN32.D3 and N38-2C12, and the Vα5^+^ (noncanonical) mouse CD1d-specific hybridoma, N37-1A12, have been described [26–28] and were cultured in IMDM supplemented with 5% FBS, 2 mM L-glutamine, in the absence of antibiotics. Purified and biotinylated monoclonal antibodies (mAb) specific for mouse IL-2, PE rat anti-mouse CD1d mAb (1B1), a rat IgG2b isotype control mAb and a FITC-labeled mAb pan-reactive anti-mouse TCRβ were purchased from BD-Biosciences (San Diego, CA). Anti-mouse CD1d mAb [1H6; ref [25].] was conjugated with Alexa488 using a commercial kit (Molecular Probes, Carlsbad, CA). Recombinant mouse IL-2 [enzyme-linked immunosorbent assay (ELISA) standard] was obtained from PeproTech (Rocky Hill, NJ). Texas Red-labeled donkey anti-rat immunoglobulin (Ig) antiserum, FITC-conjugated rat anti-mouse Ig antiserum and PE-conjugated goat anti-rat Ig antiserum were purchased from Jackson ImmunoResearch Laboratories (West Grove, PA). PE-conjugated rabbit anti-mouse Ig antiserum was obtained from DakoCytomation (Carpinteria, CA). The FcRII blocking antibody, 2.4G2, and the pan-anti-MHC class I mAb, TIB126, were from the American Type Culture Collection (Manassas, VA). Normal rat serum was purchased from Sigma-Aldrich (St. Louis, MO). The anti-mouse CD1d1 mAbs: 1H6 [25], 1E2, 8F3, 9E4, and 1A8 generated by us have been described previously [29]. The anti-MHC class I (K^b^) exon-8 specific rabbit antiserum and human TAP1-expressing recombinant vaccinia virus (VV) [30] were kindly provided by Drs. J. Yewdell and J. Bennink (Laboratory of Viral Diseases, National Institute of Allergy and Infectious Diseases, National Institutes of Health, Bethesda, MD). HRP-conjugated anti-rabbit IgG was purchased from Biorad (Hercules, CA).

### Generation of bone marrow-derived dendritic cells (BMDCs)

BMDCs were generated as previously described [31]. Briefly, bone marrow cells were harvested and cultured in media (RPMI 1640 supplemented with 10% FBS, 2 mM L-glutamine, 50 µM 2-mercaptoethanol and antibiotics) containing 10 ng/ml murine GM-CSF and 10 ng/ml IL-4 (PeproTech) for seven days.

### Treatment of cells with citric acid buffer

Acid treatment of cells was done as described previously [32]. Briefly, LMTK-CD1d1 cells, LMTK-control cells, BMDCs, splenocytes and thymocytes were washed twice in phosphate-buffered saline (PBS) before treatment with citric acid buffer (pH 3.0) for the indicated lengths of time. The pH was then neutralized with a 20 volume excess of ice cold IMDM supplemented with 10% FBS, 2 mM L-glutamine and penicillin/streptomycin, washed two more times in the same medium and fixed for 10 min at room temperature in 1% paraformaldehyde in PBS for analysis by flow cytometry or 0.05% paraformaldehyde for co-culture experiments, followed by two washes. It is important to note that sufficient quenching of acidic buffer is necessary immediately following the stripping treatment to prevent significant cell death. Following treatment an aliquot of cells was stained with trypan blue and counted to assess viability to insure equal numbers of live cells were used for subsequent analyses.

### NKT cell assays

To measure endogenous antigen presentation by CD1d, mock- or citric acid buffer-treated LMTK-CD1d1 and LMTK-control cells or other antigen presenting cells were washed, fixed in 0.05% paraformaldehyde, washed and then co-cultured (5×10^5^ cells/well) with the NKT cell hybridomas, DN32.D3, N38-2C12 or N37-1A12 [26–28] (5×10^4^ cells/well) in triplicate wells of 96-well microtiter plates. After a 24 h co-culture, supernatants were harvested, and IL-2 production was measured by ELISA as previously described [25]. For blocking assays, 5 µg/ml of anti-CD1d mAb (1H6), the anti-MHC class I mAb TIB126 or isotype control mAbs were added to mock- or citric acid-treated LMTK-CD1d1 or LMTK-control cells for 1 h on ice. The cells were then washed and co-cultured with the NKT cell hybridomas as described above.

### Flow cytometry

All cells were fixed in 1% paraformaldehyde, treated with the purified anti-mouse CD1d mAbs 1H6, 1E2, 8F3, 9E4, 1A8, the rat pan-anti-mouse MHC class I mAb TIB126 or respective isotype control mAbs [25,29], and then stained with a PE-conjugated rabbit anti-mouse Ig antiserum or PE-conjugated goat anti-rat Ig antiserum. The FcRγ II molecules on splenocytes and thymocytes were blocked by the 2.4G2 mAb before being stained with a PE-conjugated rat anti-mouse CD1d (1B1), PE-conjugated K^b^-specific mAb, Alexa488-labeled anti-mouse CD1d (1H6) or appropriate isotype control mAb. Analysis was performed by flow cytometry as previously described [25]. For quantitative analysis of acid stripping-induced changes, the geometric mean for each Ab in the mock treated cells from each assay was normalized to 100. The geometric mean of the acid stripped cells was then converted to the percent of the mock treated cells. NKT cells were identified by staining with an allophycocyanin-conjugated CD1d1 tetramer loaded with α-galactosylceramide (α-GalCer) (NIH Tetramer Core Facility, Atlanta, GA) and co-stained with anti-TCRβ.

### Confocal microscopy

Confocal microscopic analysis was performed as previously described [33]. Briefly, LMTK-CD1d1 cells were plated in sterile glass-bottom 35-mm dishes coated with collagen (MatTek, Ashland, MA). After overnight adherence, the cells were washed and fixed in 4% paraformaldehyde for 10 min at room temperature. Excess paraformaldehyde was quenched using a 10 mM PBS-glycine solution. Cells were permeabilized with 0.1% saponin (Sigma-Aldrich) in HBSS/BSA (permeabilizing buffer). MHC class I staining was performed by incubating the cells with TIB126, followed by a Texas Red-conjugated donkey anti-rat Ig antiserum. After blocking the free antibody-reactive sites with normal rat serum, immunofluorescent localization of murine CD1d1 molecules was performed by incubating the cells with 1H6, followed by a FITC-conjugated donkey anti-mouse Ig antiserum. Cell nuclei were stained with Hoechst (5 µg/ml, Molecular Probes) for 10 min in permeabilizing buffer. All Abs were diluted and incubated in permeabilizing buffer and cells were treated for 45 min at room temperature. After each step of Ab incubation, the dishes were washed three times in permeabilizing buffer. For analysis, the cells were placed in mounting medium (10 mM Tris and 2% 1-4-diazobicyclo[2.2.2] octane) and stored in HBSS/BSA+azide in the dark at 4^o^C until confocal analysis. Cells were viewed with a Zeiss confocal laser-scanning microscope equipped with a UV-laser. The FITC and Texas Red emissions were recorded sequentially using a 63X water immersion lens.

### Co-immunoprecipitation

KT4-CD1d1, KT4-CD1d1TD and KT4-control cells (or BMDCs) were washed in PBS and resuspended in ice-cold lysis buffer [58 mM Tris (pH 7.4), 173 mM NaCl, 10 mM EDTA, 0.02% sodium azide, 1.0% CHAPS, 1mM sodium orthovanadate and 1 mM sodium fluoride (phosphatase inhibitors)] containing a protease inhibitor mix (Complete Mini, Roche, Indianapolis, IN) for 30 min at 4^o^C. The lysate was precleared overnight with mouse serum-bound protein A sepharose beads (Pierce, Rockford, IL). Total protein (300 µg) was immunoprecipitated with 10 µg of purified 1H6 or isotype control mAb bound to protein A sepharose beads overnight. The beads of the bound immunoprecipitate were washed five times in PBS. To the beads was added 30 µl of 2X reducing sample buffer; proteins were resolved on a SDS-PAGE gel and transferred to a polyvinylidene difluoride membrane (Millipore, Billerica, MA). The blot was probed with a MHC class I (K^b^) exon 8-specific rabbit antiserum followed by an anti-HRP rabbit Ab and developed using chemiluminescence before exposure on film. Five µg of total lysate from each cell line and control cells (C1R, human B-cell lymphoblastoma; kindly provided by Dr. S. Balk, Harvard University) were used as positive and negative controls in Western blot analyses. Quantitation of the relative band intensity was determined by ImageJ software (National Institutes of Health, Bethesda, MD).

### Statistical analysis

All experiments were performed at least twice and representative experiments are shown with error bars representing the SEM unless otherwise noted. Multiple groups were compared by a one-way ANOVA with a Bonferroni post-test using GraphPad PRISM software (version 5.0 for Windows; GraphPad, San Diego, CA). Two groups were compared using an unpaired two-tailed t test. A *P* value below 0.05 was considered significant.

## Results and Discussion

### MHC class I molecules inhibit CD1d-mediated Ag presentation

**Figure 1:**
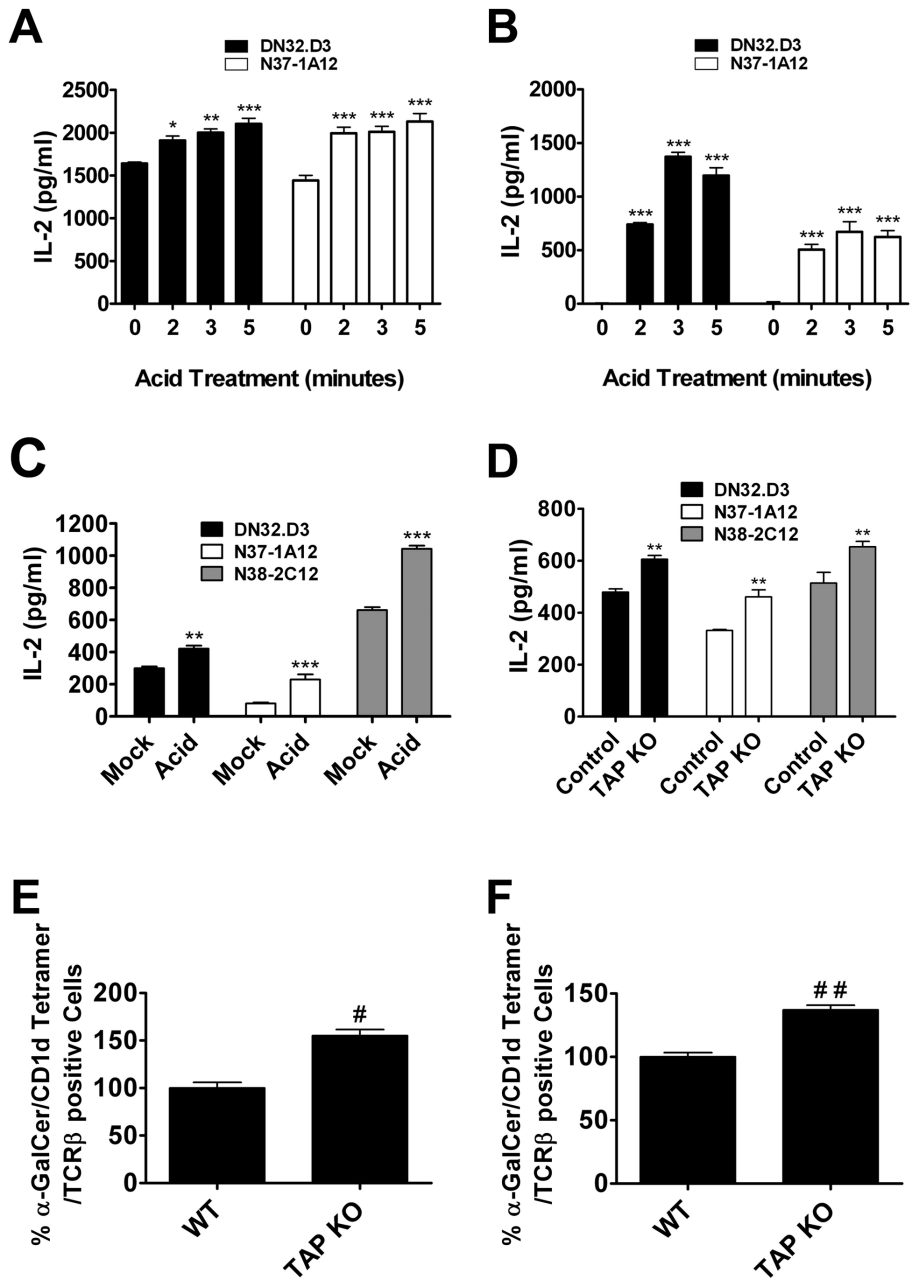
Disruption of cell surface MHC I molecules enhances CD1d-mediated Ag presentation. (A–C) Antigen presenting cells (A) LMTK-CD1d1, (B) LMTK-control or (C) bone marrow-derived dendritic cells (BMDCs) were mock-treated or treated with citric acid phosphate buffer, washed, fixed and co-cultured with NKT cell hybridomas. Treatment of APCs in acidic conditions resulted in an increase of CD1d-mediated antigen presentation; * P < 0.05, ** P < 0.01, *** P < 0.001 compared to control. (D) BMDCs generated from WT control and TAP1-/- mice were co-cultured with the indicated NKT hybridomas; ** P < 0.01. (E and F) (E) Splenocytes and (F) liver mononuclear cells were purified from TAP1-/- mice or WT controls. Type I NKT cells were identified through staining with APC-conjugated CD1d tetramers loaded with α-GalCer and FITC-conjugated anti-TCRβ mAb; # P = 0.025, # # P = 0.0172.

To analyze the role of MHC class I molecules in the regulation of the functional expression of CD1d in APCs, we stripped off cell surface MHC class I molecules using low pH citric acid buffer [32]. Treatment of cells with acidic buffer denatures MHC class I, as detected by Ab binding, and strips off any bound peptides. Cells expressing exogenous mouse CD1d were treated with citric acid buffer for increasing amounts of time, and CD1d-mediated Ag presentation was analyzed by co-culturing the cells with NKT cell hybridomas. Disruption of MHC class I molecules resulted in an increase in Ag presentation by CD1d as shown by increased IL-2 production by NKT cells upon stimulation by acid-stripped LMTK-CD1d1 cells (Figure 1A). Interestingly, LMTK-vector control cells were able to stimulate IL-2 production from NKT cells following acid stripping (Figure 1B). This suggests that there is a low level of endogenous CD1d in these cells, but under normal conditions it is not sufficient to elicit an NKT cell response. In line with this idea, there were undetectable levels of CD1d as assessed by flow cytometry pre-acid treatment (data not shown). To confirm these findings in professional APCs, murine BMDCs were either mock-treated or treated with acid buffer. As was observed with LMTK-CD1d1 and LMTK-vector control cells, BMDCs also showed an increase in CD1d-mediated Ag presentation upon acidic buffer treatment (Figure 1C). These results suggest that MHC class I is a negative regulator of CD1d-mediated Ag presentation and this inhibitory effect can be diminished by denaturing MHC class I.

Transporter associated with antigen presentation 1-deficient (TAP1-/-) mice show greatly reduced expression of cell surface MHC class I molecules [34]. Thus, TAP1-/- mice may be a good model for analyzing the effects of reduced cell surface MHC class I molecules on the function of other antigen presenting molecules such as CD1d. BMDCs from TAP1-/- mice were moderately more efficient in stimulating NKT cells compared to BMDCs from WT controls (Figure 1D). There was no significant difference in the cell surface expression of CD1d between these mice (data not shown). To analyze the status of NKT cells in these mice, liver mononuclear cells (LMNCs) and splenocytes were isolated from TAP1-/- and wild type (WT) control mice. A higher percentage of type I NKT cells (α-GalCer loaded CD1d tetramer and TCRβ double positive cells) were found in the spleen and liver of TAP1-/- mice compared to those of WT controls (Figure 1E, F).

A previous report by Joyce and colleagues also showed an expansion of NKT cells in TAP-1-/- mice [35]. In this work, they suggest that this increase in NKT cells is due to expression of the Thymus Leukemia (TL) Antigen. Our results suggest an alternative explanation for the increased NKT cell population observed in these mice: TAP-1-/- mice have more NKT cells due to increased functional expression of CD1d. It is interesting that this prior report and our current study suggest that surface MHC class I may be able to modulate the functional expression of multiple MHC class I-like molecules.

It has been shown previously that MHC class I molecules, through binding to Ly49, deliver inhibitory signals to NKT cells [36]. Dendritic cells required incubation with an anti-Ly49 mAb or the BMDCs had to be derived from TAP1-/- mice for CD1d to effectively present α-GalCer to Ly49+ NKT cells [36]. Thus, it is possible that the cause for the observed increase in the NKT cell population in TAP1-/- mice could have been due to the lack of MHC class I/Ly49 or other NK cell receptor(s) interactions, and not necessarily due to increased CD1d-mediated Ag presentation. However, there are differences between that study and the results presented here. In these experiments, we used NKT cell hybridomas as compared to NKT cells freshly isolated from mice; hybridoma generation (and long term culture of fresh NKT cells) results in a down-regulation of NK cell receptor expression [37,38]. Thus, the experiments described in the current report allowed us to focus on NKT cell TCR/CD1d interactions exclusively. It has not been reported whether Ly49 expression is retained on the hybridoma cells (although it is highly unlikely for the reasons indicated above) or if it has any functional control over activation. We did find that even on mock-treated cells that have high MHC class I levels, the NKT cell hybridomas are able to be stimulated to secrete IL-2. This is in contrast to the prior report [36], where Ly49 had to be absent or blocked to detect IFN-γ production [36]. Thus, the current study suggests that the presence of cell surface MHC class I molecules might be generating inhibitory signals to NKT cells via antigen presenting cells by masking CD1d expression and not just through Ly49 interaction, because removal of MHC class I molecules stimulated NKT activation. The presence of MHC class I molecules on the cell surface may actually impair the functional expression of CD1d molecules, and in turn NKT cell activation, through an alternative mechanism: physical interaction with CD1d. It is likely that *in vivo* there are multiple mechanisms by which MHC class I can affect CD1d-mediated Ag presentation to NKT cells, suggesting a complex regulation of the CD1d/NKT cell axis.

### Increased NKT cell activation following acid stripping is CD1d-dependent

**Figure 2:**
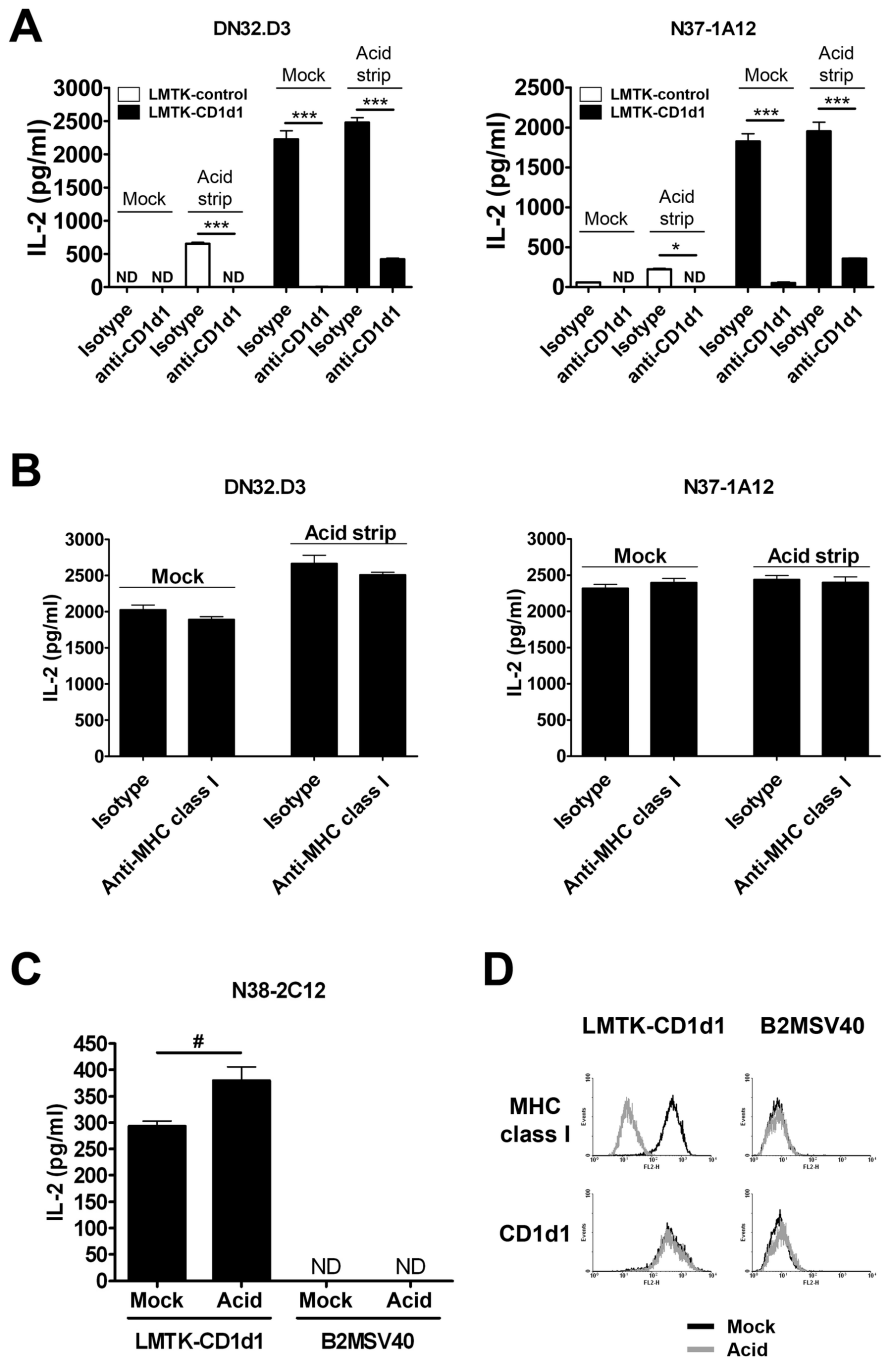
Stimulation of NKT cells by acid-treated antigen presenting cells is CD1d-dependent. (A) Mock-treated or acid-stripped LMTK-control or LMTK-CD1d1 cells were incubated with a CD1d-specific mAb (1H6) or an isotype control mAb. LMTK-control or LMTK-CD1d1 cells incubated with the 1H6 mAb showed reduced CD1d-mediated Ag presentation to NKT cells; * P < 0.05, *** P < 0.001, ND = not detectable. (B) Mock-treated or acid-stripped LMTK-CD1d1 cells were incubated with a pan-MHC class I-specific mAb (TIB 126) or an isotype control mAb. Incubation with TIB 126 or an isotype control did not alter CD1d-mediated Ag presentation. (C) Mock- or acid-treated LMTK-CD1d1 and B2MSV40 cells were co-cultured with NKT hybridoma cells. LMTK-CD1d1 (but not B2MSV40) cells showed increased stimulation of NKT cells following acid treatment; # P = 0.0356, ND = not detectable. (D) Mock-treated and acid-stripped LMTK-CD1d1 and B2MSV40 cells were stained with the 1H6 and TIB 126 mAbs to detect surface CD1d1 and MHC class I, respectively.

In order to show that enhanced CD1d-mediated Ag presentation in acid treated-cells is actually CD1d-dependent, acid-treated or untreated LMTK-control or LMTK-CD1d1 cells were incubated with the CD1d-specific mAb 1H6 or an isotype control, and then co-cultured with NKT cell hybridomas. Incubation with the CD1d-specific mAb 1H6, but not with the isotype control mAb, blocked the stimulation of NKT cells by both untreated and acid-treated LMTK-control or LMTK-CD1d1 cells (Figure 2A). Additionally, the incubation of untreated or acid treated LMTK-control or LMTK-CD1d1 cells with the MHC class I-specific mAb TIB126 did not alter the stimulation of NKT cells (Figure 2B). This confirms that enhanced stimulation of NKT cells by acid-treated antigen presenting cells is CD1d-specific.

Although the blocking mAb 1H6 was able to completely abolish increased NKT stimulation by acid-treated LMTK-control cells, there was still a low level of cytokine production from LMTK-CD1d1 cells. It is likely that due to the high levels of CD1d expression on these cells, mAb blocking was incomplete. However, it was also possible that there is some CD1d-independent activation. To test this, we used B2MSV40 cells derived from β2m-deficient mice that do not express surface CD1d1. As seen in Figure 2C, acid-stripped LMTK-CD1d1 cells showed increased NKT cell activation compared to mock-treated cells. On the other hand, the B2MSV40 cells failed to stimulate NKT cells even after acid stripping. This further suggests that NKT cell activation following acid treatment is CD1d-dependent. A clear reduction can be seen in surface MHC class I on LMTK-CD1d1 cells, whereas surface CD1d (1H6-reactive) levels were unchanged following acid stripping (Figure 2D). B2MSV40 cells do not show surface MHC class I or CD1d or a change following treatment with acid.

### Acid stripping results in increased surface CD1d

**Figure 3:**
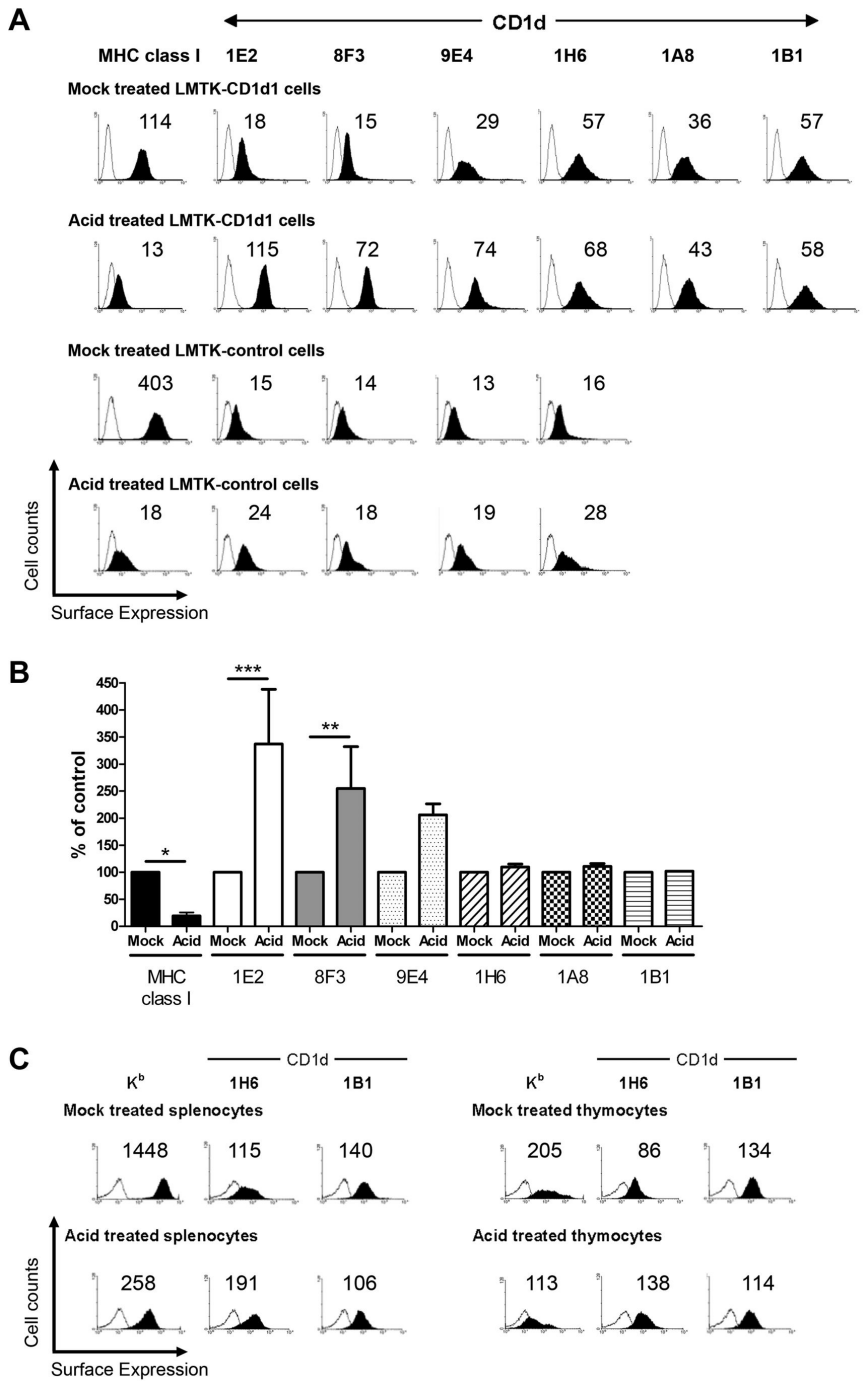
Low pH-mediated disruption of cell surface MHC I molecules enhances cell surface CD1d expression. (A) Cell surface MHC class I (K^k^) and CD1d expression on mock-treated LMTK-CD1d1 (row 1), acid-treated LMTK-CD1d1 (row 2), mock-treated LMTK-control (row 3), and acid-treated LMTK-control (row 4) cells were stained using a pan-murine MHC class I mAb, a panel of mouse CD1d-specific mAbs or their respective isotype control mAb. (B) Changes in MHC class I and CD1d surfaces levels after acid stripping is dependent on the antigen and Ab used. Combined results of multiple experiments with the geometric mean of the mock-treated cells for each mAb normalized to 100; * P < 0.05, ** P < 0.01, *** P < 0.001. (C) Cell surface MHC class I (K^b^) and CD1d expression on mock-treated (row 1) and acid-treated (row 2) splenocytes (left) and thymocytes (right) from C57BL/6 mice were measured. The numbers above each histogram indicate the mean fluorescence intensity. Open histogram: isotype control; filled histogram: CD1d- or MHC class I-specific staining.

As seen in Figure 2D, there is a decrease in surface MHC class I but no change in surface CD1d following stripping with acid. To detect CD1d in these experiments, only the 1H6 mAb was used. Thus, it was important to further investigate how CD1d-mediated Ag presentation is enhanced in MHC class I-stripped cells, whether it is due to quantitative and/or qualitative changes in the functional expression of cell surface CD1d. Thus, mock- or acid treated cells were stained for cell surface CD1d with a panel of anti-CD1d antibodies. Acid-treated LMTK-control and LMTK-CD1d1 cells showed enhanced expression of cell surface CD1d (Figure 3A). LMTK-control cells have very low to undetectable cell surface CD1d levels under normal conditions, but treatment of these cells with acidic buffer resulted in increased cell surface CD1d (Figure 3A). The combined results from multiple experiments for LMTK-CD1d1 cells are quantified in Figure 3B. Treatment of cells with acid significantly decreased the amount of MHC class I on the surface as expected. Changes in CD1d1 surface expression were dependent on the Ab used for detection, as three showed an increase and three showed minimal changes. Staining with 1E2 and 8F3 showed significant increases following acid treatment. 9E4 staining showed over a 2-fold increase, although it was not statistically significant. Conversely, staining with 1H6, 1B1 and 1A8 mAbs did not show any significant changes in surface CD1d. This suggests that some epitopes on CD1d are changed by acid-treatment, whereas others are not. An alternative explanation could be that some epitopes are blocked by MHC class I binding to CD1d and this blockage is removed by denaturing MHC class I with acid. The specific CD1d1 epitopes that these antibodies recognize are not known. Biochemical and biophysical studies that are beyond the scope of this report would be needed to distinguish between these possibilities.

As with LMTK-control and LMTK-CD1d1 cells, splenocytes and thymocytes also showed an increase in cell surface expression of CD1d upon treatment with acidic buffer, with the exception of the 1B1 mAb (Figure 3C). As expected, acid treatment almost completely reduced cell surface staining for MHC class I molecules (Figure 3C). Thus, the increased stimulation of NKT cells by acid-treated cells was likely due to quantitative changes in the surface expression of CD1d. It could be envisaged that the denaturing of MHC class I molecules by acidic buffer exposed additional CD1d molecules on the surface that had been previously masked by binding to MHC class I. The resultant effect would allow increased CD1d-specific mAb binding and enhanced CD1d-mediated Ag presentation to NKT cells in acid-treated, as compared to mock-treated CD1d^+^ cells.

### CD1d and MHC class I molecules physically interact

**Figure 4:**
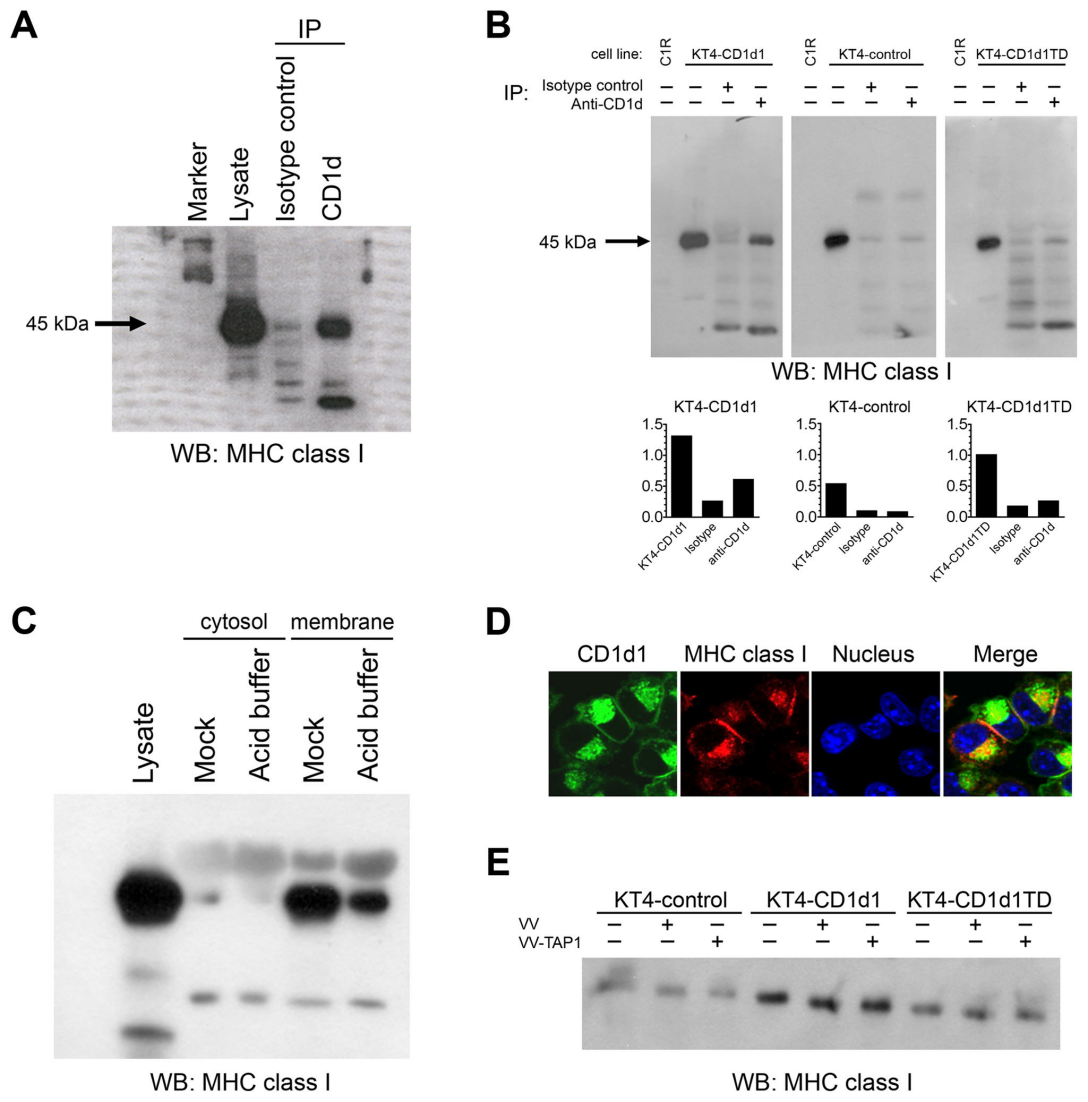
Murine MHC class I and CD1d molecules physically associate. (A and B) Lysates from BMDCs (A) or mock-treated and acid-stripped KT4-CD1d1, KT4-CD1d1TD, or KT4-control cells (B) were co-immunoprecipitated with the 1H6 mAb or an isotype control mAb and immunoblotted with an anti-MHC class I mAb. Presence of a MHC class I–specific band in the lane loaded with lysate co-immunoprecipitated with the CD1d-specific mAb 1H6, but not in the lane loaded with lysates treated with an isotype control mAb, shows the association of MHC class I and CD1d molecules. The graphs below (B) indicate relative band intensities. (C) Mock- or acid-treated KT4-CD1d1 cells were lysed to prepare membrane and cytosolic fractions; the association of MHC class I and CD1d was analyzed in both fractions. (D) Confocal microscopy shows co-localization of CD1d (green) and MHC class I (red). (E) KT4-CD1d1, KT4-CD1d1TD and KT4-control cells were mock-infected or infected with a control VV or VV-TAP1 for 4 h. Cells were lysed and co-immunoprecipitated with the 1H6 mAb and immunoblotted with an anti-MHC class I mAb.

MHC class I molecules have been reported to be physically associated with MHC class II molecules at the cell surface [39]. Although CD1d is structurally similar to MHC class I, its intracellular trafficking is similar to MHC class II molecules. CD1d and MHC class II molecules have also been found to be associated both at cell surface and in endocytic compartments [40,41]. To understand the mechanism of how CD1d-mediated Ag presentation is altered by the acid stripping of cell surface MHC class I, we asked whether CD1d and MHC class I molecules form a complex. We looked for the association of CD1d and MHC class I molecules in BMDCs by immunoprecipitating CD1d and performing a Western blot to detect MHC class I. CD1d and MHC class I molecules were indeed found to be associated in BMDCs (Figure 4A).

Next, we wanted to examine whether the intracellular tail of CD1d is required for this interaction. We used KT4 cells [24] because they lack endogenous CD1d expression, thereby allowing us to express WT and tail-deleted CD1d. Additionally, KT4 cells are derived from a TAP1-deficient mouse. Due to the lack of TAP1 expression, these cells express lower levels of MHC class I [24]. Such an approach reduced the possibility of non-specific interactions with CD1d. KT4-control or KT4-CD1d1 cells were lysed and immunoprecipitated using the CD1d-specific mAb, 1H6 [25]. Western blotting with an anti-MHC class I-specific antiserum (anti-K^b^ exon 8) demonstrated the physical association of CD1d and MHC class I molecules (Figure 4B). Lysates from human C1R cells were included as a negative blotting control. These results demonstrate that not all MHC class I and CD1d molecules are physically associated. This likely explains why even under mock conditions, there is some CD1d detectable by the antibodies that show increased binding following acid stripping. The CD1d tail plays an important role in the recycling and Ag loading of CD1d to the cell surface, as cells containing a tail deleted (TD) form of CD1d are poorly recognized by NKT cells [25,42]. To understand the importance of the CD1d tail in the CD1d/MHC class I complex, lysates from KT4 cells expressing CD1d1TD were used in a co-immunoprecipitation assay. Unlike in KT4-CD1d1 cells, the association of CD1d and MHC class I was found to be weak in KT4-CD1d1TD cells (Figure 4B). However, co-immunoprecipitation of MHC class I was not completely abolished when the tail deleted form of CD1d was used. This suggests that although the tail contributes to the binding between CD1d and MHC class I, other regions of CD1d are likely also involved.

To show that the association of MHC class I and CD1d is occurring in the membrane, mock- or acid-treated KT4-CD1d1 cells were fractionated into membrane and cytosolic fractions as previously described [31]. Both membrane and cytosolic fractions from mock- or acid-treated cells were immunoprecipitated with the 1H6 anti-CD1d mAb and Western blotted with the anti-K^b^ exon 8 antiserum. Membrane fractions from mock-treated cells showed a higher level of CD1d/MHC class I association as compared to membrane fractions from acid-treated cells (Figure 4C), whereas cytosolic fractions from cells receiving either treatment did not show any substantial association of MHC class I and CD1d (Figure 4C). Confocal microscopy was used to further assess the association of CD1d and MHC class I molecules. CD1d and MHC class I molecules clearly co-localized; this was particularly apparent intracellularly, very likely the endoplasmic reticulum (Figure 4D). Although it is not possible to say that this colocalization is the same as a physical interaction, it is important that these molecules are in close proximity inside of cells, as this suggests that the interaction is not just an artifact of cell lysis.

Because KT4 cells are deficient in TAP1, it may be questioned that absence of TAP1 molecules might be affecting the association of MHC class I and CD1d. To rule out this possibility, KT4-control or KT4-CD1d1 or KT4-CD1d1TD cells were either mock-, control vaccinia virus (VV) or VV-TAP1-infected. The association of MHC classes I and CD1d was not altered in any of these cells upon VV-TAP1 infection (Figure 4E). This suggests that TAP1 does not play an important role in the cell association of MHC class I with CD1d.

In summary, these data clearly show that MHC class I physically associates with CD1d and regulates its functional expression on the cell surface. NKT cells secrete large amounts of cytokines upon stimulation with lipid-loaded CD1d molecules. Unregulated stimulation of NKT cells may contribute to hyperactivation and exacerbated autoimmune diseases. Thus, for the host, it is important to keep the activity of immune cells in balance. MHC class I molecules are not only crucial in immunity against viruses and tumors, but also regulate the innate arm of the immune system through the physical association with CD1d and consequent regulation of CD1d-mediated Ag presentation. Thus, even when MHC class I is down-regulated during conditions such as certain viral infections, CD1d is still able to present antigens and can do so to an even greater extent. This could allow for an immune response via cytokine release from NKT cells and subsequent recruitment and activation of other immune cells. Additionally, there is the possibility that this interaction between CD1d and MHC class I molecules could affect the ability of MHC class I to present antigen. We have not explored this here, but it would certainly be interesting to examine if there is reciprocal regulation between CD1d and MHC class I in subsequent studies.
